# Influence of tillage systems and sowing dates on the incidence of leaf spot disease in *Telfairia occidentalis* caused by *Phoma sorghina* in Cameroon

**DOI:** 10.1038/s41598-022-23920-4

**Published:** 2022-11-17

**Authors:** Kebei Andrew Kpu, Mbong Grace Annih, Agyingi Lucy Ambang, Ebile Dayan Agwah

**Affiliations:** 1 Department of Plant Biology, Faculty of Science, University of Dschang, Santchou, Cameroon; 2 Department of Animal Science, Faculty of Agronomy and Agricultural Sciences, University of Dschang, Santchou, Cameroon

**Keywords:** Ecology, Plant sciences

## Abstract

The production and leaf quality of *Telfairia occidentalis* in Cameroon are gravely threatened by leaf spot disease. A field study was conducted in July 2019 and 2020 to determine the influence of tillage systems and sowing dates on the incidence of *Telfairia occidentalis* leaf spot caused by *Phoma sorghina*. A randomized block design with three replications and four planting dates was used. The soil physicochemical properties were also determined. Data for disease incidence were registered once every two weeks and submitted to generalized linear model for statistical analysis. The means were separated by Duncan’s multiple range test at a 95% confidence interval. Calculations for disease incidence and statistical analysis were conducted using the Microsoft Excel program and SPSS version 23, respectively. Statistical analysis revealed that the zero tilled field generally registered a lower leaf spot disease incidence than the tilled field, which was highly significant (*p* ≤ 0.05). In addition, the second sowing date in the zero tilled field proved to be better in terms of low disease incidence than other sowing dates employed in the study. Therefore, to minimize the devastating effects of leaf spot disease, zero tillage coupled with the second sowing date could be exploited.

## Introduction

*Telfairia occidentalis* Hook. f. is a perennial leafy vegetable of the cucurbit family^1^. The plant is known primarily in Cameroon for its dark green leaves and has been reported to be of immense nutritional, medicinal and economic benefit to the human population and even beyond^2,3^. It grows on all soil types particularly in well-drained soils and produces an annual yield of leaves and vines of 8.29 Mt. ha^−1^, leaves 5.52 Mt. ha^−1^ and seed 2.08 Mt. ha^−1^. The harvesting of leaves is usually between 120 and 150 days after sowing^4^.

The immense potential of the crop is largely threatened due to diseases and pests^5^. Leaf spot disease caused by a pathogenic fungus, *Phoma sorghina*, has been reported to be the most devastating threat to the production of *T. occidentalis*^5^. The visible effects of the pathogen on the leaves are small circular translucent white spots. The spots enlarge, turn brown and shatter, leaving the leaves with perforations. Under severe attack, the entire leaf dies^6^. Foliar lesions of dead or collapsed cells are produced as the pathogen infects the leaves, which in turn reduces the leaf lamina's thickness. This has the effect of limiting production and degrades its quality, thereby reducing its market value and profitability. When the pathogen infects leaves, the nutrient content is drastically reduced^6^.

Hence, management of the disease is of paramount importance to safe guard yields in Cameroon. Different disease management techniques have been reported in the control of leaf spot disease. Nwufo and Ihejirika^7^ reported that fungicides can be used in the management of the disease under field conditions. The authors intimated that spraying with fungicides every fortnight had the effect of significantly minimizing disease incidence in the field. Nevertheless, chemical control of plant diseases is of potential danger to humans, nontarget beneficial organisms and the environment^6^ and can also result in residual chemicals in food^8^. As a disease management strategy, the use of chemicals cannot be ruled out, but their high cost and long-term effects on the natural ecosystem remain disturbing^9^. In addition, these chemicals leave nonbiodegradable residues in the soil. These chemicals are not only scarce but are also very expensive to poor farmers who even lack expertise and other accessories in the usage of these chemicals^6^. These methods have therefore been ineffective in the management of the disease.

Therefore, evaluation of other cultural methods to control leaf spot disease, such as soil tillage and planting date, could help to develop alternative strategies. In Cameroon, *T. occidentalis* is cultivated on zero-till soil and on till soil on mounds. To safeguard sufficient supplies of the leafy vegetable during the dry season, when the demand is very high, many farmers tend to set up production farms at different planting dates in July. Ibeawuchi et al.^10^ reported that demand for *T. occidentalis* is throughout the year, with a significant rise in the dry season, when the market value of produce is very high. In some cases, farmers resort to cutting down or removing any existing vegetation, followed by punching and sowing without tilling the soil, in an incessant quest for maximizing time. Other farmers, however, resort to tilling the soil and constructing mounds before sowing. Some studies have been conducted on the role tillage practices play in the growth and yield of *T. occidentalis*^11^. The crop performs well in terms of vegetative growth in tilled and zero-tilled soils. Okeke et al.^11^ reported that, *T. occidentalis* planted on zero-tilled plots competed in terms of growth rate with *T. occidentalis* planted on mound-tilled plots. Nahoua et al.^12^ reported that, sowing date is important in tropical climates. Crop plant diseases can be avoided or controlled by using sowing date-based strategies, according to the authors. There is, however, a dearth of information on how to use a specific tillage system and planting dates in Cameroon to manage *T. occidentalis* leaf spot disease, which is unfortunate. Cultivators are therefore completely unaware of how the commonly employed soil tillage systems and planting time could be masterminded in the management of leaf spot disease. This is compounded by the fact that reliable data are largely unavailable and the literature on these management techniques is very scant.

In this study, we attempted to evaluate the effectiveness of different soil tillage systems and sowing dates in the management of leaf spot disease in *T. occidentalis*.

## Materials and methods

### Area of study

The field study was set up in the locality of Santchou within the farming seasons of 2019 and 2020 at the Institute for Agricultural Research and Development (IRAD), research and seed multiplication field in July.

Santchou is located between 5°16′N and 9°58′E. It has an altitude of 786 m with a surface area of 95.05 km^2^. The annual average temperature in Santchou is 22.5 °C. Its annual average precipitation is 1,364.4 mm with a relative humidity of 92%^13^. Santchou has very complex vegetation, and its climate is equatorial to the Guinean type (Santchou council development plan, 2015), similar to the Littoral and Southwest regions, which are hosted to several cultivators of *T. occidentalis*. Rains are heavier from mid-August to October and lower from March to June^13^. The study area is characterized by two main seasons: the dry season, which runs from mid-November to March, and the rainy season, which runs from March to November.

### Experimental design

The experiment for the two years was laid out in a randomized block design (RBD), with 36 experimental units laid out in three blocks. The factors were two tillage systems and four sowing dates. Within each block, three experimental units were selected randomly and sowed for each of the four separate sowing dates. The experiment was laid out over a surface area of 121 m^2^. Each experimental unit measured 2.25 m^2^. The experimental units and blocks were both separated by alleys of 0.4 m. The experimental layout for each planting season was the same.

### Soil sample analysis

Just before the study was conducted, soil samples were collected randomly from the aid of a soil auger at depths of 0–20 cm from the topsoil. The soil samples were scrupulously mixed to obtain a composite sample and taken to the laboratory for physicochemical analysis prior to field preparation. The soil physical and chemical components were all determined at the Laboratory of Soil Science and Environmental Chemistry of the Faculty of Agronomy and Agricultural Sciences (FASA), University of Dschang. The procedure for soil sampling analysis was conducted with the aid of the publication of^14^.

The soil particle size was determined by using the hydrometer method as described by Rowell^15^. The soil pH was determined using a glass electrode pH meter in a 1:2.5 soil to water ratio as described by Almaz et al.^16^. The cation exchange capacity (CEC) was determined as a direct continuation of exchangeable base determination as described by^17^.

The amount of available phosphorus in the soil samples was determined using the Bray 2 method described by^17^. The total nitrogen was determined by making use of the Kjeldahl method^18^.

The percentage organic matter in the two soil samples was taken as an average of 58% organic carbon. Its content was calculated from the percentage of organic carbon as shown in the following equation:

$$ {\text{Percentage organic matter }} = {\text{ percentage organic carbon}} \times 1.724 $$

where 1.724 is a cofactor.

The soil organic carbon was determined by Walkley and Black’s method^19^.

### Planting materials

Intact and mature *T. occidentalis* fruit pods for seeds, harvested from senescent shoots from an intercropped research farm of these authors in the town of Dschang^5^, were used as planting material. A minimum number of fruit pods of the same cultivar required for the research were harvested. To extract the seeds, pods were cut open with the aid of a knife, and the seeds were carefully isolated from the pulp manually. The seeds, spread out on a dry surface, were air dried for two days to prevent decay before sowing. The seeds used in the subsequent year were of the same cultivar. The experimental research and field studies of the cultivated crop plant, including the collection of planting material were within institutional, national and international guidelines and legislation.

### Field preparation

Separate fields each measuring 121 square meters at the IRAD research field in Santchou were manually cleared of the weeds. The cleared debris was removed and dumped beyond the field experiment. In one of the fields, a hoe was later employed to uniformly plow the field to facilitate the construction of regular experimental units. A decameter, pegs and cords were exploited to demarcate the tilled field into experimental units, and hoe was again made use of to set up mounds of 1.5 m × 1.5 m separated by passage ways of 0.4 m.

In the second field, blocks and experimental units were delineated into three blocks comprising 36 experimental units. The dimensions were all similar to those obtained in the tilled field, and a decameter, pegs and strong thick cords were used to facilitate the process. In both tilled and no-tilled fields, plots were identified with labeled tags to distinguish each experimental unit as per the sowing date for a methodical and quality disease assessment and ideal follow-up in the field.

### Sowing of seeds

The topsoil was used as substrate for sowing. Healthy air-dried seeds were taken to the field and sown by direct seeding at a depth of 3–4 cm and covered with topsoil on each experimental unit at a rate of 1 m × 1 m. Four seeds were sown per experimental unit.

For each year, four sowing dates were chosen and staggered seven days apart to determine the most appropriate time that sowing the crop produces minimal leaf spot disease incidence. The four sowing dates were July 4th, 11th, 18th, and 25th for the first, second, third and fourth sowing dates respectively.

### Crop maintenance in the field

After the four-week sowing string, the field was constantly monitored for weed removal and staking with advancing growth. The removal of weeds commenced two weeks after emergence in the field. This was achieved manually once every fortnight to ensure optimal growth voids of other plant competitors and for better monitoring of disease parameters in the field.

Staking with the aid of pegs locally harvested in the vicinity of the field was initiated at three weeks after emergence and continued for an additional period of two weeks. The pegs were trimmed to a height of 1 m. The staked stands were tied with robes drawn from plantain stems. This was meant to train the clambering vines to the trellis and to facilitate their creeping pattern. Bamboo trellises were constructed for each experimental unit to serve as a supporting platform for optimal crop growth and for ideal disease assessment.

### Data collection

#### Disease assessment in the field

Disease incidence in the field was established by visual observations of symptoms of *T. occidentalis* leaf spot on the leaves on all *T occidentalis* stands in the treatments. Data collection for disease incidence commenced three weeks after emergence when typical symptoms of the disease begin to appear and continued for eight weeks at two weeks interval. In the process, diseased leaves and the totality of leaves for each stand were counted, and the information was meticulously documented.

#### Calculations for *disease* incidence

The information recorded in the field was used to calculate the percentage of the proportion of leaves infected per plant within the speculated period using the modified formula for disease incidence by^20^.

$$ {\text{Disease incidence }} = \frac{{\text{Number of leaves infected per plant}}}{{\text{Total numberof leaves sampled}}} \times 100 \% $$

#### Statistical analysis

The information documented on the total number of leaves and number of diseased leaves from each stand at the corresponding sowing date was submitted to generalized linear model for analysis and the means were separated by Duncan’s multiple range test (DMRT) at a 95% confidence interval. The disease incidence was calculated using the Microsoft Excel program, while SPSS was used for ANOVA.

### Ethical standard

We state that this research complies with ethical standards.

## Results and discussion

### Results

#### Soil physiochemical properties

The preliminary status of the soil analyzed before the commencement of the field preparatory activities revealed that the soil was subtlety fertile with regard to the physical and chemical properties (Table 1).

**Table 1:** Physicochemical properties of the soil.

Parameter	Property
pH_water_	6.0
pH_KCl_	5.4
Organic carbon (%)	5.42
Organic matter (%)	9.35
Nitrogen (g/kg)	3.27
C/N	17
Calcium (meq/100 g)	3.68
Magnesium (meq/100 g)	12.32
Potassium (meq/100 g)	0.31
Sodium (meq/100 g)	0.16
Sulphur (meq/100 g)	16.47
Phosphorus (mg/kg)	16.28
CEC (meq/100 g)	25.37
Sand (%)	48
Silt (%)	24.5
Clay (%)	27.5

#### Assessment of disease incidence at sowing dates during each year in the trial study

In the trial study, very low and statistically significant (*p* < 0.05) disease incidences were recorded at sowing date one in the second year in both the tilled and zero tilled fields. On the contrary, very high and statistically significant leaf spot incidences were registered at the first and third sowing date in the tilled field during the first year of the trial investigation (Table 2).

**Table 2:** Assessment of disease incidence at sowing dates in the trial study.

Sowing date	Tillage system	DI (%) ± SE	DI (%) ± SE
		2019	2020
July 04	Till	43.46 ± 1.48^a^	9.46 ± 1.48f
	Zero till	37.00 ± 1.48^b^	13.66 ± 1.48^e^
July 11	Till	38.15 ± 1.48^b^	14.19 ± 1.48^e^
	Zero till	19.47 ± 1.48^d^	7.12 ± 1.48f
July 18	Till	47.22 ± 1.48^a^	19.77 ± 1.48^d^
	Zero till	29.80 ± 1.48^c^	20.22 ± 1.48
July 25	Till	30.79 ± 1.48^c^	15.82 ± 1.48^de^
	Zero till	29.68 ± 1.48^c^	16.54 ± 1.48^de^

*SE* Standard Error; *DI* Disease incidence.

a, b, c, d, e, f means in the columns with the same superscript are not significantly different at *p* < 0.05 (DMRT).

#### Assessment of disease incidence between the tillage systems during each year of the study

In the investigation, extremely low and statistically significant (*p* < 0.05), leaf spot disease incidences were documented in both the tilled and zero tilled fields in the first and second year of the study. Contrarily, the tilled field in 2019 recorded a very high leaf spot incidence which was significantly different from the incidences registered in zero tilled field in the same year as well as from both tillage systems in the second year of the field study (Table 3).

**Table 3:** Disease incidence in the tillage systems.

Tillage system	Year	Disease incidence (%) ± SE
Till	2019	39.90 ± 0.74^a^
Zero till	2019	28.99 ± 0.74^b^
Till	2020	14.81 ± 0.74^c^
Zero till	2020	14.39 ± 0.74^c^

*SE* Standard Error.

a, b, c means in the same column with the same superscript are not significantly different at *p* < 0.05 (DMRT).

#### Assessment of disease incidence during both years of the investigation

The study revealed that, the first year of the study (2019), recorded a higher *T. occidentalis* leaf spot incidence than the incidence observed in the second year. Statistical analysis revealed that the disease incidences differed significantly (Table 4).

**Table 4:** Disease incidence in years of the study.

Year	Disease incidence (%) ± SE
2019	34.45 ± 0.52^a^
2020	14.60 ± 0.52^b^

*SE* Standard Error.

^a, b, c^ means in the same column with the same superscript are not significantly different at *p* < 0.05 (DMRT).

#### Mean leaf spot disease incidence within the tillage systems

In the trial investigation, the zero tilled field globally recorded a lower and statistically significant leaf spot disease incidence than was observed in the tilled field (Table 5).

**Table 5:** Mean leaf spot disease incidence in the tillage systems.

Tillage system	Disease incidence (%) ± SE
No till	21.69 ± 0.52^b^
Till	27.35 ± 0.52^a^

*SE* Standard Error.

^a, b^ means in the same column with the same superscript are not significantly different at *p* < 0.05 (DMRT).

#### Mean leaf spot incidence at sowing dates

The field experiment divulged that, the second sowing date in the untilled field comprehensively recorded a very *T. occidentalis* low leaf spot incidence which was significant (*p* < 0.05), and differed statistically from other incidences investigated in the study. By and large, the disease incidence was rife and statistically significant at sowing date three within the tilled field (Table 6).

**Table 6:** Mean leaf spot disease incidence at sowing date.

Sowing date	Tillage system	Disease incidence (%) ± SE
One (July 04)	Till	26.46 ± 1.05^b^
	Zero till	25.33 ± 1.05^b^
Two (July 11)	Till	26.17 ± 1.05^b^
	Zero till	13.29 ± 1.05^d^
Three (July 18)	Till	33.49 ± 1.05^a^
	Zero till	25.01 ± 1.05^b^
Four (July 25)	Till	23.30 ± 1.05^b^
	Zero till	23.11 ± 1.05^c^

*SE* Standard Error.

^a, b, c, d^ means in the same column with the same superscript are not significantly different at *p* < 0.05 (DMRT).

### Discussion

The study established the vulnerability of *T. occidentalis* to leaf spot disease under field conditions. The nitrogen content of the soil was found to be very high. Adequate nitrogen levels are necessary for disease resistance. However^21^, explained that excess nitrogen may promote favorable conditions for plant disease. The authors argued that excess nitrogen promotes thinner and weaker cell walls and delays the maturity of plant tissues and therefore increases the risk of disease infection and development. Ekwere et al.^22^ reported that the total percentage nitrogen recommended by^23^, as the critical value for good crop production, is 2%. The nitrogen in the soil was therefore in excess. *T. occidentalis* stands were more susceptible to disease because of the excess nitrogen in the soil, except for the second sowing date, when the disease incidence was high. The prevalence of leaf spot disease was further compounded in the tilled field, which was characterized by a more humid microclimate compared to the zero-tilled experimental units.

In addition, the amount of phosphorus in the soil available for the crop was high. However, its role in resistance is variable and seemingly inconsistent. Jones et al.^24^ reported that increasing phosphorus rates above the level needed for plant growth increased the prevalence of *Fusarium* wilt in cotton and muskmelon. During the first, third, and fourth sowing dates of this study, a high phosphorus content may have contributed to the development of leaf spot disease in the field.

The findings in this study revealed that the tilled and zero-tilled fields during the second year of the investigation registered lower and statistically significant leaf spot disease incidences than the tillage systems in the previous year. This could be due to the fact that, the environmental temperatures were lower in 2020 than in 2019 (Tables 7 and 8). Thus, lower temperatures, together with higher rainfall and higher relative humidity could have been significant in reducing leaf spot disease incidence in the field irrespective of the tillage system.

**Table 7:** Average monthly climatic parameters in Santchou 2019. Source: IRAD DSCHANG.

	January	February	March	April	May	June	July	August	September	October	November	December
Average temperature (°C)	28	28	28	27	26	23	22	22	23	23	23	26
Rainfall (mm)	94.9	94.9	94.9	508.63	508.63	508.63	873.1	873.1	873.1	369.97	369.97	369.97
Humidity (%)	17.7	17.7	17.7	25.0	25.0	25.0	28.1	28.1	28.1	24.3	24.3	24.3

**Table 8:** Average monthly climatic parameters in Santchou 2020. Source: IRAD DSCHANG.

	January	February	March	April	May	June	July	August	September	October	November	December
Average temperature (°C)	27	28	26	25	24	22	21	21	21	22	24	25
Rainfall (mm)	194.3	194.3	194.3	614.7	614.7	614.7	874.1	874.1	874.1	386.83	874.1	874.1
Humidity (%)	21.2	21.2	21.2	27.2	27.2	27.2	28.3	28.3	28.3	26.1	26.1	26.1

Generally, in the field investigation, the zero tilled field registered a low leaf spot incidence compared to the tilled field which was significantly different.

This result is unique in its kind and innovative in the management of leaf spot disease in *T. occidentalis*. Previous investigations by^25^ obtained other results. Similar observations were reported by^26^ Soil tillage has been shown to have advantages by^23^; however, in this study the growing stages of the stands in the tilled field may have been more susceptible to infection by *Phoma sorghina* coupled with more encouraging microclimatic conditions.

Santchou is characterized by a terrain that is low land and extensively flat, which makes drainage following intense precipitation within this period virtually impossible, if not difficult. As the rainy period progressed and became increasingly intense, the land became inundated. This period coincided with the short season when the study was being conducted. It was impossible to avoid flooding during this time because of the lack of drainage channels due to the flatness of its terrain, which caused water levels to rise significantly. In effect, the field was submerged in water. In addition, the soil became completely soaked and soggy, with the result that the microclimate of the field was modified with conditions rendered more humid and sustained throughout the short season. A buildup of these uninterrupted humid conditions could have been particularly significant where the field was tilled. This observation is consistent with the findings of^27^, who reported that the high incidence of scab and anthracnose was probably due to a relatively humid microclimate, which favors epidemics of these diseases. Soil tillage loosens the soil, creating pores and spaces for water to readily seep and flood the topsoil. It's possible that the leaf spot pathogen multiplied quickly due to the high and persistent humidity, and since the growing stages of the crop were vulnerable, new infections were unavoidable due to the high and persistent spore production and the susceptibility. As a result, the tilled field saw an increase in the incidence of leaf spot disease.

Furthermore^28^, reported that species of the fungi pathogen are widely distributed in the environment, most commonly found in aquatic systems and soil. There could have been a large number of inoculums in the heavily flooded field, and the weather conditions were favourable for the pathogen to prevail and infect the plants. Earlier findings by^29^ and later^30^ confirmed that the optimum temperature for the growth of mycelial spores of *Phoma sorghina* lies in the range of 20–25 °C. The average temperature in Santchou that prevailed within the study period was within the range of 20–25 °C, and it is probable that this temperature highly favored conidial germination and further multiplication of the pathogen within a very short time period. To put it another way, a large inoculum density, coupled with the vulnerability of perennial vegetables, could have resulted in the high disease incidence that was observed in tillage fields.

The first and second sowing dates in the second year of the study within the tilled and zero tilled fields respectively registered very low and statistically significant leaf spot incidences. Helen and Michele^31^ reported that changing the usual sowing time of a crop can exploit weather conditions that are not favorable for the spread of pathogens and reduce crop losses due to diseases caused by pathogenic microorganisms.

Globally in the study, the second sowing date registered an extremely low and statistically significant *T. occidentalis* leaf spot disease incidence. This result, in this study, revitalizes the fact that, adjustment of sowing dates is strategic in the prevalence of crop diseases under field conditions. Previous investigations by^32^, revealed similar results. The author reported with empirical evidence that disease incidence was significantly affected by different planting dates.

The findings in this study confirm the holistic aspect (tillage systems and adjustment of sowing dates) of the management of plant diseases by creating an environment more suitable for plant growth but not for disease development. The second sowing date within which *T. occidentalis* leaf spot disease incidence was minimal could have coincided with growing stages of the crop that were less susceptible, more resistant, to infection and spread of the leaf spot pathogen, resulting in leaf spot disease avoidance. The results in this study are also in agreement with previous investigations carried out and reported by^33^. The researchers affirmed that the adjustment of planting dates is an important cultural practice that can be exploited to minimize crop losses due to disease. According to the authors, powdery mildew severity in sunflower decreased as a result of a strategic sowing date manipulation. The authors ascertained that such a cultural technique avoided coincidence with the susceptible stage of the crop, consequently resulting in disease escape. Subsequent reports by^34^ confirmed that strategic alteration in planting dates was effective in the control of some plant diseases. Previous reports^35,36^, established that sowing dates significantly influenced the epidemiology of crop diseases under field conditions. The observations established in this study and in other empirical studies, as reported by^30–36^, further confirm the fact that this cultural disease management technique, manipulation of planting time, is vital in reducing food crop diseases under field conditions.

The very high disease incidence observed on the third planting date in the tilled field could be due to a more favorable microclimate and very high vulnerability of the growing stages of crop plants to infection. Therefore, the more conducive microclimate coupled with a conceivably high initial inoculum population could have encouraged the proliferation of the already populated fungal spores and their germination and rapid multiplication, which favored new and rapid infections, resulting in extremely high leaf spot disease incidences and severities. Earlier studies by^12^ found that disease spread was aided in cucurbits under field conditions by humid and warm weather conditions. In addition^37^, acknowledged that leaf spot diseases are favored by humid weather conditions, where they destroy a greater portion of the foliage. Humid conditions are required for spore germination. With warm and massively humid conditions, as was the case in this study, the spores readily germinated within a brief period of time, resulting in further spread of the disease among the more vulnerable stands.

## Conclusion

Generally, in the study, the zero tilled technique proved better in reducing *T. occidentalis* leaf spot disease. In addition, the second sowing date (July 11) investigated in this study was significant in minimizing the disease in the field in Santchou. These cultural disease management strategies, which are accessible, applicable, inexpensive, and safe to humans, can be embraced by underprivileged cultivators to enhance the production of *T. occidentalis* leaves at profitable levels.

## Data Availability

The datasets generated and/or analyzed during the current study are available from the corresponding author on reasonable request.
